# Predictors of Intestinal Parasitic Infection among Food Handlers Working in Madda Walabu University, Ethiopia: A Cross-Sectional Study

**DOI:** 10.1155/2020/9321348

**Published:** 2020-01-17

**Authors:** Kemal Ahmed Kuti, Rameto Aman Nur, Geroma Morka Donka, Amene Abebe Kerbo, Adem Esmael Roba

**Affiliations:** School of Health Science, Madda Walabu University, P.O. Box 302, Bale-Robe, Ethiopia

## Abstract

**Background:**

Intestinal parasitic infection is one of the major health problems globally. It is more common in developing countries including Ethiopia. So, adequate evidence is needed regarding the predictors of intestinal parasitic infection. This study was aimed at determining the predictors of intestinal parasitic infection among food handlers working in Madda Walabu University, Ethiopia.

**Methods:**

An institution-based cross-sectional study was conducted from 15 May to 10 June 2017 among 198 symptom-free food handlers. Data on sociodemographic variables were collected through face-to-face interview using a structured questionnaire. Stool samples were collected immediately after the interview using labeled wide-mouthed plastic container and clean wooden applicator. Direct wet-mount method and formal-ether concentration techniques were performed to identify intestinal parasites. The data were analyzed using SPSS version 21.0. Descriptive statistics and crude and adjusted odds ratios with 95% confidence interval (CI) were calculated. *p* value of <0.05 was considered to declaration level of significance.

**Result:**

The response rate was 98% (198/202). The overall prevalence of intestinal parasites was 25.3% (50/198). The top three intestinal parasites found in this study were *Ascaris lumbricoides* 7.6% (15/198), *Entamoeba histolytica/dispar* 7.6% (15/198), and hookworm 5.6% (11/198). Inadequate handwashing practice (AOR: 13.876; 95% CI: 4.129, 46.632), inadequate knowledge about foodborne diseases (AOR: 3.596; 95% CI: 1.438, 8.989), lack of training on proper food handling (AOR: 5.960; 95% CI: 1.450, 24.497), and untrimmed fingernail (AOR: 2.939; 95% CI: 1.368, 6.135) were independent predictors of intestinal parasitic infection.

**Conclusion:**

High prevalence of intestinal parasites was observed among symptom-free food handlers who could be unobservable source of disease transmission. Inadequate handwashing, untrimmed fingernail, inadequate knowledge, and lack of training were independent predictors of intestinal parasitic infection in this study. This implies the need for timely and adequate training and enforcement of regular medical checkup system for food handlers.

## 1. Introduction

Food is highly susceptible to contamination unless the strict hygienic procedure is followed. Its contamination can occur at any point during its journey starting from production through processing, distribution, and meal preparation. There are various factors which may increase the risk of contamination of food; some of them are health status of the food handlers, their personal hygiene, knowledge, and practice of food hygiene [1].

Health problems related to food are one of the most crucial problems that affect public health globally [2]. It was estimated that about 30% of the population living in the developed world suffered from diarrheal diseases which were mostly caused by foodborne microbial pathogens. About 2 million deaths occur annually due to foodborne diseases in developing countries. Foodborne diseases are also common in higher education institutions in Ethiopia [3–5].

As a result of urbanization and eating and drinking from common food establishments, which is becoming a common practice in developing countries, the chances of suffering from foodborne diseases are increasing. The health status and hygienic practices of food handlers are the major determinants of food contamination. In developing countries where there are poor regulatory systems for food hygiene, food handlers are often appointed without screening for possible infections associated with poor hygiene like intestinal parasites [4].

The diarrheal diseases which are caused by foodborne or waterborne microbial pathogens are known to be the leading causes of morbidity and mortality in underdeveloped world, resulting in an estimated 1.9 million deaths annually at the global level. Even in developed countries, an estimated one-third of the populations suffer from microbiological foodborne diseases annually [6].

The intestinal parasites can be protozoan or helminths living within the human body. They are more common in tropical and subtropical areas of the world [7, 8]. Intestinal parasitic infection is one of the major health problems globally. About 3.5 billion people are infected, and around 450 million people are ill due to intestinal parasites worldwide [9]. Most of the time, intestinal parasitic infections do not show clinical signs and symptoms and also have a number of potential carriers, such as food handlers, which makes it too difficult to eradicate and control [10]. The problem of infection with intestinal parasites is higher in developing countries including Ethiopia due to lack of adopting optimal hygienic practices during food handling [2, 10–12]. Nearly more than half of the cases of diarrheal disease in developing countries are linked with the consumption of foods contaminated with intestinal parasites [2].

The factors associated with infection of intestinal parasites that were identified by previous studies include fingernail status and handwashing practice [4, 13], having training certificate on food handling [14], being female, rural residence and socioeconomic status [15], and educational status [16]. Higher education institutions are places where a large number of students and workers are served from the same food sources that are handled by some food handlers. This shows the need for timely and adequate scientific evidence about the status and predictor factors of intestinal parasitic infection among food handlers as there may be asymptomatic carriers who may affect a large number of individuals. On top of that, the context of higher education institution may be a very necessitating need for specific information in order to be able to act accordingly. Thus, this study was aimed at assessing the status and predictors of intestinal parasitic infection among food handlers working in Madda Walabu University.

## 2. Methods

### 2.1. Study Design and Study Setting

An institution-based cross-sectional study was conducted from 15 May to 10 June 2017. The study participants were food handlers who were working in food establishments at Madda Walabu University. The university is located in Bale-Robe Town in the Oromia Regional State of Ethiopia at 430 km to southeast direction from the capital city of the country, Addis Ababa. It has an additional campus at Goba Town which is located at a distance of 14 km from Bale-Robe Town where the main campus is located.

### 2.2. Study Participants

The total number of food handlers working in the university was two hundred fourteen (214) only. As a result, all of them were considered for this study. However, about 12 food handlers who were ineligible because of being on treatment for intestinal parasitic infection or due to other severe illnesses were excluded from the study. The remained 202 participants were selected and included in this study.

### 2.3. Data Collection and Specimen Processing

A structured questionnaire was prepared by reviewing various literature studies related to the study. It was prepared in English and translated to local languages (Afan Oromo and Amharic) and then retranslated to English to check for its consistency. The questionnaire and specimen collection processing were pretested on 5%, among food handlers working in private food and drinking establishment of Bale-Robe Town to check for clarity and consistency. After pretest, the observation checklist was added at the end of the questionnaire and the questionnaire was finalized. The data collectors were given training for two days on data collection tool, data collection procedure, specimen collection, specimen processing, and research ethics. They were also participated in the pretest process.

Data about the study variables were collected by two Bachelor of Science (BSc) degree holder nurses through face-to-face interview technique. At the end of the interview, the study participants were linked to medical laboratory professionals for stool specimen provision. The specimen collection and processing was done by two BSc degree holder medical laboratory professionals following the standard procedure. Stool specimen was collected by providing labeled wide-mouthed plastic container and clean wooden applicator to each participant and instructing them to bring about 5 g of their own stool. The specimen was transported using icebox to microbiology laboratory of the university immediately within 15 minutes of collection for processing. The stool specimen of each participant was examined by direct wet-mount method and formal-ether concentration techniques according to the standard procedures recommended by the World Health Organization [17]. Initially, each stool specimen was examined for its consistency and categorized as formed, soft, loose, or watery. Then, each specimen was examined using saline wet mount (0.85% NaCI solution) to identify protozoan trophozoites and cyst as well as eggs and larvae of worms. Each stool specimen with cyst of protozoa was examined with iodine wet mount (using Lugol's iodine). Buffered methylene blue (BMB) wet mount was used when protozoan trophozoites are suspected or identified. The remaining specimen was preserved with 10% formalin for further examination of protozoan morphology and further development of helminth egg and larvae. The investigators did close follow-up of overall activities throughout the data collection and specimen processing period.

### 2.4. Data Analysis

The data collected using questionnaire and laboratory investigations were checked manually, entered into Statistical Package for Social Science (SPSS) version 21, cleaned, and made ready for further analysis. The data were checked for accuracy, consistencies, missed values, and presence of any outliers. Descriptive statistics such as frequency, percentage, and mean were calculated. The association between dependent and each independent variable was checked by applying binary logistic regression, and then, the variables with *p* value of <0.25 were fit into multiple logistic regression through backward conditional model in order to check for the presence of an independent association between dependent and independent variables. Multicollinearity was checked among the independent variables using variance inflation factor (VIF). The assumptions of logistic regression were checked using Hosmer and Lemeshow goodness-of-fit test statistics. The result was reported using adjusted odds ratio (AOR) with 95% confidence interval (CI). *p* value of less than or equal to 0.05 was taken for the declaration of the presence of statically significant association.

## 3. Results

### 3.1. Participants

**Figure 1:**
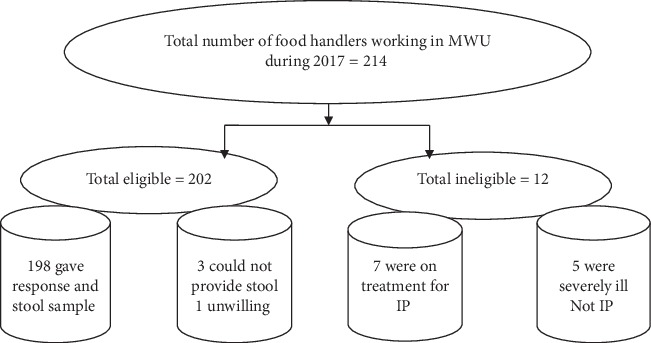
A flow diagram showing the selection and recruitment process of food handlers working in food establishment of Madda Walabu University, Bale-Robe, Ethiopia, 2017. IP = intestinal parasites.

A total of 198 (98%) of 202 food handlers who were eligible for this study gave response. The remaining 4 participants did not respond because 3 of them could not provide stool sample after repeated trial while one participant was unwilling to participate (Figure 1).

### 3.2. Sociodemographic Characteristics

The mean age of study participants was 25.95 ± 7.538 (95% CI: 24.88, 27.00). About half (51.5%) of the participants were in the age range of 18–24 years, while the majority (66.2%) of them were females. About 46% of them were single and nearly half (51%) of them attended only primary school (grades 1–8). About 37.4%, 17.1%, 27.8%, and 17.7% were cook, utensil cleaners, waiters, and other service providers, respectively. 37.4% of the respondents had less than six months of service time as food handler while 23.2% had greater than three years of work experience as food handler (Table 1).

### 3.3. Hygiene-Related Issues

The respondents were assessed about their handwashing practice using six questions that were based on self-report. The overall level of handwashing practice was classified as adequate practice when correct responses are given for all questions and otherwise classified as inadequate practice. Accordingly, 179 (90.4%) had adequate handwashing practice. 57 (28.8%) and 38 (19.2%) of them were trained on food handling and had certificate of training, respectively. The study participants were observed for use of personal protective devices during food handling. Accordingly, 41 (20.7%) covered their hair and wear gown even though only 29 (14.6%) of the gowns were clean and 104 (52.5%) of the participants had trimmed their fingernail (Table 2).

### 3.4. Knowledge of Participants on Foodborne Diseases

The study participants were assessed for their knowledge about foodborne diseases by asking eleven questions comprising of the types of foodborne diseases, things that play role in transmission of foodborne disease, and methods of prevention. Regarding types of foodborne diseases, about 33 (16.7%), 131 (66.2%), 84 (42.4%), 52 (26.3%), and 94 (47.5%) of the respondents mentioned ascariasis, typhoid fever, amebiasis, giardiasis, and acute watery diarrhea, respectively. With regard to things that play role in transmission of foodborne diseases, contaminated fluids, contaminated finger, fly, lack of hygiene, and feces were mentioned by 120 (60.6%), 110 (55.6%), 58 (29.3%), 50 (25.3), and 43 (21.7%) respondents, respectively.

Different methods of prevention of foodborne diseases were mentioned. Accordingly, 171 (86.4%), 69 (34.8%), and 17 (8.6%) of the participants had mentioned personal hygiene, optimal cooking of food, and having regular medical checkup, respectively. The knowledge level of the respondents was categorized as adequate knowledge or inadequate knowledge based on the mean score. That is, the respondents who scored above mean were categorized as having adequate knowledge while those who scored less than or equal mean were categorized as having inadequate knowledge about foodborne diseases. Accordingly, 75 (37.9%) of the participants had adequate knowledge while 123 (62.1%) had inadequate knowledge about foodborne disease.

### 3.5. Prevalence of Intestinal Parasitic Infection

**Figure 2:**
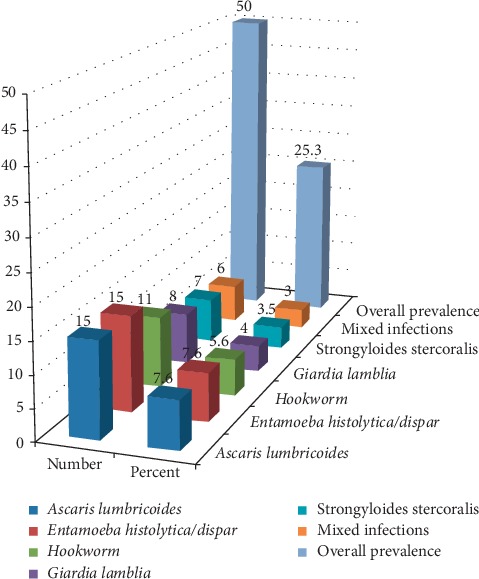
Prevalence of intestinal parasitic infection among food handlers working in food establishment of Madda Walabu University, Bale-Robe, Ethiopia, 2017.

Out of the total of one hundred ninety-eight (198) stool samples investigated, intestinal parasites were seen in 50 (25.3%) of the stool samples. Regarding the specific type of intestinal parasite identified, *Ascaris lumbricoides*, *Entamoeba histolytica*/*dispar*, hookworm, *Giardia lamblia*, and *Strongyloides stercoralis* were identified in 15 (7.6%), 15 (7.6%), 11 (5.6%), 8 (4%), and 7 (3.5%), respectively. Mixed infections were found among 3% (6/198) of study participants (Figure 2).

### 3.6. Predictors of Intestinal Parasite Infection

During bivariable logistic regression analysis, inadequate handwashing practice (COR: 11.122; 95% CI: 3.760, 32.901), inadequate knowledge about foodborne diseases (COR: 3.133; 95% CI: 1.457, 6.733), lack of training on food handling (COR: 3.450; 95% CI: 1.152, 10.213), and untrimmed fingernail (COR: 3.520; 95% CI: 1.768, 7.007) showed statistically significant association with the presence or absence of intestinal parasite at *p* value <0.05. However, in multivariable regression analysis, only handwashing practice (AOR: 13.876; 95% CI: 4.129, 46.632), inadequate knowledge about foodborne diseases (AOR: 3.596; 95% CI: 1.438, 8.989), lack of training on food handling (AOR: 5.960; 95% CI: 1.450, 24.497), and untrimmed fingernail (AOR: 2.939; 95% CI: 1.368, 6.315) remained independent predictors of intestinal parasitic infection (Table 3).

## 4. Discussion

Infection with intestinal parasites could be transmitted through food and water contamination resulting from improper food handling by carrier persons working in food and drinking establishments [18]. It is still one of the major health concerns in developing countries which require understanding of risk factors for the design of effective intervention strategies [8]. So this study was aimed at assessing the magnitude of intestinal parasites and its associated factors among food handlers working in Madda Walabu University food establishments.

Out of the total sample of two hundred two, one hundred ninety-eight participants responded to the questionnaire and provided stool sample resulting in a response rate of 98% (198/202). The overall prevalence of intestinal parasites in this study was 25.3%. This result is in close agreement with studies conducted among food handlers at the University of Gondar and Gondar Teachers Training College, Ethiopia, 29.1% [19]; Eldoret Town, Kenya, 30.5% [18]; and Gaza Strip, Palestine, 24.3% [20].

On the other hand, the result of this study is lower than studies conducted in Addis Ababa University, Ethiopia, 45.3% [3]; Yebu Town, Southern Ethiopia, 44.1% [4]; Bahir Dar Town, Northwest Ethiopia, 41.1% [6]; and Arba Minch University, Southern Ethiopia, 36% [13]. But the result of this study revealed higher prevalence of intestinal parasite than studies conducted in Aksum Town, Northern Ethiopia, 14.5% [12]; Tabriz City, Northwestern Iraq, 4.7% [16]; Khorramabad Town, western Iraq, 9% [14]; and Bagalkot City, India, 14.7% [13].

The possible reason for the differences observed could be due to the difference among study population (employees versus general population) and the differences in setting, the differences in the educational and other socioeconomic conditions, and the differences in hygiene of the individuals and the working environment.

This study showed that the most prevalent intestinal parasites among study participants were *Ascaris lumbricoides* and *Entamoeba histolytica/dispar* 7.6% each, followed by hookworm 5.6%, *Giardia lamblia* 4%, and *Strongyloides stercoralis* 3.5%. Regarding the most prevalent intestinal parasites, this study agreed with studies conducted in Arba Minch University, Southern Ethiopia, which reported 14% *Entamoeba histolytica/dispar* and 9.27% of *Ascaris lumbricoides* [13]; in Bahir Dar Town, Northwest Ethiopia, which reported 11.7% of *Ascaris lumbricoides* and 12.76% of *Entamoeba histolytica/dispar* [6]; in the University of Gondar and Gondar Teachers Training College, Northwest Ethiopia, which reported 18.11% of *Ascaris lumbricoides* [19]; in Yebu Town, Southern Ethiopia, which reported *Ascaris lumbricoides* as predominant parasite [4]; in Bagalkot City, India, which reported *Ascaris lumbricoides* and *Entamoeba histolytica/dispar* 3.5% and 1.5%, respectively [15]; and study conducted in Gaza Strip, Palestine, which reported 19.2% of *Entamoeba histolytica/dispar* [20].

The observed similarity may be due to the easily communicability nature of these parasites. This study differed from studies conducted in Tabriz City, Northwestern Iraq, in which case *Giardia lamblia* was the prevalent parasite [16], and a study conducted in western Iran [14]. The difference may possibly be explained by the difference in eating habits, climatic conditions, and other sociocultural differences between the study setting and participants.

Regarding predictors of intestinal parasitic infection, inadequate handwashing practice (AOR: 13.876; 95% CI: 4.129, 46.632), lack of knowledge about foodborne diseases (AOR: 3.596; 95% CI: 1.438, 8.989), lack of training on proper food handling practice (AOR: 5.960; 95% CI: 1.450, 24.497), and untrimmed fingernail (AOR: 2.939; 95% CI: 1.368, 6.315) were independent predictors of intestinal parasitic infection. Regarding the association of intestinal parasitic infection with fingernail status and handwashing practice, this study is in agreement with studies conducted in Arba Minch University, Southern Ethiopia [13], and Yebu Town, Southern Ethiopia [4]. It also agreed with the study done at Khorramabad Town, western Iraq [14].

But it was differed regarding the association between the presence of intestinal parasites and knowledge about foodborne diseases and training on proper food handling practice. The observed difference may be explained by the difference in the level of education and level of training between the study populations. The current study also differed from the study conducted in Bagalkot City, India, which reported female sex, rural residence, and socioeconomic status having association with intestinal parasites [15], and in Tabriz City, Northwestern Iraq, which reported association with educational status [16]. This might be due to the difference in socioeconomic structure/status and educational status between the study participants of this study and other studies.

The result of this study implied that policymakers, healthcare officials, higher education administrators, and individuals directly or indirectly involved in food handling should play their role in strengthening of training and regular medical checkup system for food handlers.

The following limitations were found in this study: *Entamoeba histolytica and Entamoeba dispar* were not identified separately. The parasite carriage of the fingernail contents and parasite intensity were not assessed in this study. The prevalence of intestinal parasites was reported based on a single stool sample examination. The study was also subject to all limitations of cross-sectional studies.

## 5. Conclusion

High prevalence of intestinal parasites was observed among symptom-free food handlers who could be an unobservable source of disease transmission. Inadequate handwashing, lack of fingernail hygiene, lack of knowledge on foodborne diseases, and lack of training on food handling were independent predictors of intestinal parasitic infection. This implies that timely and adequate training should be given for food handlers in the form of preservice and on-service training together with enforcement of regular medical checkup by food handlers.

## Figures and Tables

**Table 1:** Sociodemographic results of food handlers working in food establishment of Madda Walabu University, Bale, Ethiopia, 2017 (*n* = 198).

S. no	Variables		Frequency	Percent
1	Age categories	18–24	102	51.5
		25–31	55	27.8
		32–38	22	11.1
		39–52	19	9.6

2	Sex categories	Female	131	66.2
		Male	67	33.8

3	Marital status	Single	91	46
		Married	70	35.4
		Divorced	30	15.1
		Widowed	7	3.5

4	Educational status	Grades 1–8	101	51
		Grades 9–12	74	37.4
		College/university	23	11.6

5	Participants job position	Cook	74	37.4
		Utensil cleaners	34	17.1
		Waiters	55	27.8
		Other services	35	17.7

**Table 2:** Hygiene practice among food handlers working in food establishment of Madda Walabu University, Bale, Ethiopia, 2017 (*n* = 198).

S. no.	Variables		Frequency	Percent
1	Handwashing always after toilet visit	Yes	192	97
		No	6	3

2	Handwashing always after contact with unclean materials	Yes	190	96
		No	8	4

3	Handwashing always before food handling	Yes	196	99
		No	2	1

4	Handwashing always after food handling	Yes	190	96
		No	8	4

5	Handwashing always before touching food utensils	Yes	192	97
		No	6	3

6	Handwashing always after cleaning utensils	Yes	188	94.9
		No	10	5.1

7	Overall handwashing practice	Adequate practice	179	90.4
		Inadequate practice	19	9.6

8	Had training on food handling	Yes	57	28.8
		No	141	71.2

9	Had certificate of training on food handling	Yes	38	19.2
		No	160	80.8

10	Did wear cap	Yes	41	20.7
		No	157	79.3

11	Wear gown	Yes	41	20.7
		No	157	79.3

12	Wear clean gown	Yes	29	14.6
		No	169	85.4

13	Trimmed fingernail	Yes	104	52.5
		No	94	47.5

**Table 3:** Result of multivariate logistic regression analysis among food handlers working in food establishment of Madda Walabu University, Bale, Ethiopia, 2017 (*n* = 198).

S. no	Variable	Categories	Intestinal parasite		AOR	95% CI		*p* value
			Yes	No		Lower	Upper	
1	Age category	18–24	25	77	0.743	0.131	4.203	0.737
		25–31	12	43	1.409	0.311	6.383	0.656
		32–38	6	16	1.075	0.179	6.474	0.937
		39–52	7	12	1			

2	Marital status	Single	22	69	0.218	0.035	1.371	0.104
		Married	15	55	0.250	0.039	1.616	0.146
		Divorced	10	20	0.409	0.056	2.988	0.379
		Widowed	3	4	1			

3	Service year	<6 months	13	61	0.483	0.161	1.451	0.195
		6 months–1 year	5	13	0.851	0.200	3.629	0.828
		1-2 years	4	12	0.479	0.084	2.747	0.409
		2-3 years	15	29	1.177	0.358	3.870	0.788
		>3 years	13	33	1			

4	Handwashing practice	Adequate	36	143	1			
		Inadequate	14	5	13.876	4.129	46.632	≤0.001

5	Knowledge about foodborne diseases	Adequate	10	65	1			
		Inadequate	40	83	3.596	1.438	8.989	0.006

6	Adequate training	Yes	4	34	1			
		No	46	114	5.960	1.450	24.497	0.013

7	Trimmed fingernail	Yes	15	89	1			
		No	35	59	2.939	1.368	6.315	0.006

## Data Availability

All datasets on which the conclusions of the manuscript rely are available from the corresponding author upon request.

## Abbreviations

AOR: Adjusted odds ratios
BMB: Buffered methylene blue
BSc: Bachelor of Science
CI: Confidence interval
COR: Crude odds ratio
IP: Intestinal parasites
MWU: Madda Walabu University
OR: Odds ratios
SPSS: Statistical Package for Social Science
VIF: Variance inflation factor
WHO: World Health Organization.
